# Predictors of Long-term Patient-Reported Outcome Measures After Collagen Meniscal Implant for Partial Meniscal Defects

**DOI:** 10.1177/23259671241254395

**Published:** 2024-07-25

**Authors:** Alberto Grassi, Gian Andrea Lucidi, Stefano Di Paolo, Andrea Pierangeli, Piero Agostinone, Giacomo Dal Fabbro, Nicola Pizza, Stefano Zaffagnini

**Affiliations:** * Clinica Ortopedica e Traumatologica II, IRCCS Istituto Ortopedico Rizzoli, Bologna, Italy; ‡ Dipartimento di Scienze per la Qualità della Vita QuVi, Università di Bologna, Italy; Investigation performed at Rizzoli Orthopaedic Institute, Bologna, Italy

**Keywords:** Collagen meniscal implant, general sports trauma, knee, meniscus, long-term follow-up, meniscal allograft transplantation, meniscal scaffold

## Abstract

**Background:**

Collagen meniscal implant (CMI) is considered an effective procedure for reducing knee pain and improving knee function after previous meniscectomy. Nevertheless, the current knowledge regarding long-term patient reported-outcome measures after CMI is limited.

**Purpose:**

To evaluate clinical outcomes, reoperations, and failures of CMI at a minimum 10-year follow-up.

**Study Design:**

Case series; Level of evidence, 4.

**Methods:**

Consecutive patients who underwent CMI at a single institution were screened for eligibility. Inclusion criteria for the present study were (1) medial or lateral CMI; (2) isolated or combined procedure with anterior cruciate ligament reconstruction, knee osteotomy, or cartilage treatment; and (3) follow-up between 10 and 15 years. Demographics and surgical details were obtained via chart review. Patients were asked if they were satisfied with the procedure and were evaluated with the Lysholm score, Knee injury and Osteoarthritis Outcome Score (KOOS), visual analog scale for pain, and Tegner score at the final follow-up. Cases requiring partial or total scaffold removal for any reason (including scaffold breakage, infection, or surgery for osteoarthritis progression) were considered surgical failure. Survival analysis was performed with Kaplan-Meier curve, and clinical scores were analyzed based on the Patient Acceptable Symptom State (PASS).

**Results:**

A total of 92 patients (mean age, 42.2 years were included in the analysis. A significant improvement in all clinical scores was reported between the preoperative evaluation and the last follow-up. A chondropathy with Outerbridge grade ≥3 was associated with significantly overall lower clinical scores, while a timing from meniscectomy to CMI of ≥5 years determined more pain at rest and reduced Quality of Life in the KOOS subscale. No significant difference was found in terms of clinical scores between patients undergoing isolated and combined procedures. At the final follow-up, the mean Lysholm score was 76.3 points. In total, 12 cases (13%) were considered surgical failures. Sixteen patients (17%) did not reach PASS for the Lysholm score, with a total of 28 cases (30%) classified as clinical failures. Overall, 19% (KOOS Pain) and 40% (KOOS Symptoms) of patients did not achieve the PASS in the KOOS subscales. Chondropathy with Outerbridge grade ≥3 was associated with a higher risk of not achieving the PASS in all the KOOS subscales, while age at surgery of ≥45 years resulted in a lower risk of not achieving PASS in the Pain subscale. At the last follow-up, 63% of patients were still involved in sports activity, with 41% at the same or higher level. Finally, 80% of the patients were satisfied with the procedure.

**Conclusion:**

Up to 10 years after surgery, around 70% of the patients who underwent CMI reported satisfactory clinical results, with clinical subjective scores still higher compared with the preoperative evaluation. Overall, 30% of cases were considered clinical failures, with 13% considered surgical failures and 17% not meeting the PASS for the Lysholm score. In addition, cartilage status and time from meniscectomy were shown to have a negative impact on the outcomes, while an age ≥45 years was associated with less pain. There was no clinical difference between patients who underwent isolated CMI or combined procedures.

Menisci play an essential role in knee biomechanics and are crucial for articular cartilage preservation.^1^ Research has shown that even a partial meniscectomy can lead to unicompartmental knee pain, recurrent swelling, and bone marrow edema in certain patients, potentially resulting in early onset osteoarthritis.^4^ This condition is also known as “postmeniscectomy syndrome.” To avoid and manage this condition, meniscal scaffold devices have been developed and proposed as an alternative to meniscal allograft transplantation (MAT) only in the case of partial meniscal loss.^4,5^

Even though it has been >20 years since the first reports on the use of meniscal scaffolds, limited literature exists on patient and surgical factors that may affect the long-term clinical outcomes of this procedure since most of the scaffold studies are limited to a short- or midterm follow-up. The few studies that evaluated the long-term outcomes of this procedure assessed mostly the failure rate and had a small sample size.^11,18,27^

Moreover, a recent systematic review evaluating the outcomes of meniscal scaffold implantation concluded that due to the presence of overlapping patients and frequent associated concomitant surgeries, most of the clinical benefit might be related to these concomitant procedures rather than the partial meniscal substitution procedures.^13^

Against this background, the primary purpose of this study was to assess the long-term clinical outcomes of the collagen meniscal implant (CMI) for partial meniscal defect, investigating if comparable results can be obtained after an isolated procedure or in combination with anterior cruciate ligament (ACL) reconstruction, knee osteotomy, or cartilage treatment. The secondary purpose was to identify possible predictors of clinical outcomes. The hypothesis was that good clinical outcomes could be obtained with CMI, both in isolated cases and in combination with other treatments. Additionally, patients with more severe chondral damage would have poorer outcomes, and delaying scaffold implantation after meniscectomy could affect clinical results.

## Methods

### Patient Selection

Institutional review board approval was obtained for the study (General protocol number: 0014969 - 02/11/2020). No specific funding was provided for the present study. All the consecutive patients who underwent CMI scaffold implantation at Rizzoli Orthopaedic Institute between 2005 and 2010 were screened. The indications for scaffold implantation and the study inclusion criteria are shown in Table 1. All the patients signed an informed consent.

**Table 1:** Indications for Surgery and Inclusion Criteria^*a*^

Indications for surgery
- Localized unicompartmental knee pain in patients with previous partial meniscectomy - Chronic bucket-handle tears not amenable for suture, chronic and complete radial tears with considerable meniscal retraction, and complex tears involving the posterior meniscal horn with tissue degeneration - Intact meniscal rim and functional anterior and posterior horn attachments - Intact ACL or an ACL-deficient knee in which an ACLR was performed at the time of CMI implantation - Absence of varus or valgus deformity or a knee in which an osteotomy was performed to correct the mechanical axis deviation if >4°
Inclusion criteria for the present study
- Medial or lateral CMI - Isolated or combined procedure with ACL reconstruction, knee osteotomy, or cartilage treatment - Follow-up between 10 and 15 years

a ACL, anterior cruciate ligament; ACLR, anterior cruciate ligament reconstruction; CMI, collagen meniscal implant.

### Patient Evaluation

Demographics and surgical details such as patient sex, CMI side (medial or lateral), body mass index (BMI), time from first meniscectomy to CMI, concurrent procedures, and cartilage status were obtained by chart review. Patients with acute irreparable tears who underwent meniscectomy and immediate CMI implantation were considered to have a time from meniscectomy to CMI of 0 years. For the purpose of the analysis, the chondropathy was stratified as “low grade” in the case of Outerbridge grades 0 to 2 and “high grade” in the case of Outerbridge grades 3 and 4.

All patients were contacted via telephone, and Knee injury and Osteoarthritis Outcome Score (KOOS), Lysholm, and Tegner questionnaires were administered. Patients were also asked to rate their knee pain at rest or during activity on a 10-point visual analog scale (VAS). Data on sport activity and participation were also investigated. Patients were asked whether they had undergone any additional surgeries on the operated knee during the follow-up period and if they were satisfied with the results of the CMI. Finally, medical records from the hospital database were screened for possible complications or further surgeries.

Surgical failures were defined as any reoperations that required a partial or total scaffold removal, including (1) infection, (2) scaffold-related complications such as CMI fragmentation or dislocation, (3) conversion to MAT, (4) unicompartmental knee arthroplasty, and (5) total knee arthroplasty. Surgical failures were not considered achieving the PASS. Clinical failures were defined as not reaching the Patient Acceptable Symptom State (PASS) for Lysholm score.

### Surgical Technique and Rehabilitation

The surgical technique for arthroscopic CMI implantation has been previously described.^11,27^ Briefly, a standard diagnostic arthroscopy was performed to confirm the indication of CMI; the ACL should be intact or concomitantly reconstructed. While diffuse grade 4 Outerbridge cartilage degeneration represented a contraindication to scaffold implant, it was treated with standard cartilage procedures such as microfracture, mosaicplasty, or scaffold implants if a focal full-thickness cartilage lesion was present. The CMI for the partial meniscal defect was implanted arthroscopically and sutured to the meniscal remnant with all-inside devices. Then, any associated procedures such as an ACL reconstruction, osteotomy, or cartilage repair surgery were performed.

A knee brace locked in full extension was applied for 6 weeks. To avoid knee stiffness, continuous passive motion was performed 4 times per day, from 0° to 60° during the first 2 weeks. The range of motion then increased to 90° from the second to the sixth week, while complete range of motion was allowed starting from the sixth week. Weightbearing was avoided for the first 3 weeks. After this period, 30% of the patient’s weightbearing was allowed during the fourth week and 50% of the patient’s weightbearing was allowed during the fifth week, with progression to full weightbearing during the sixth week. Return to sports and cutting activity was permitted starting 6 months after surgery.^27^

### Statistical Analysis

Statistical analysis was performed with MedCalc (Acacialaan 22; MedCalc software). The continuous variables were expressed as mean ± SD, while the categorical variables were expressed as numbers and percentages. A comparison among various follow-up points and between the isolated and combined groups was performed with unpaired-samples *t* test in case of continuous variables, and differences in categorical variables were analyzed with the chi-square test. The patients were also stratified according to the PASS threshold for the Lysholm score (66.5 points), KOOS Symptoms (73.0 points), KOOS Pain (43.0 points), KOOS Activities of Daily Living (KOOS ADL) (74.5 points), KOOS Sport and Recreation (KOOS Sport) (22.5 points) and KOOS Quality of Life (KOOS QoL) (53.0 points) as defined by Liu et al^15^ for meniscal transplantation. In the case of missing values for clinical scores owing to surgical failure, the preoperative values were used to avoid overestimation of the clinical status when calculating the postoperative mean values. Moreover, patients with surgical failure were considered as not having achieved the PASS for all the scores and subscales. The total of patients with surgical failure and those not reaching the PASS for the Lysholm score were defined as having experienced clinical failure. Survival analysis was performed with Kaplan-Meier curve.

Multiple regression analysis models with a stepwise approach were performed using clinical scores as endpoints and sex (male vs female), CMI side (medial vs lateral), age (<45 years vs ≥45 years),^3^ chondropathy (Outerbridge grades 0-2 vs 3-4), associated procedures (isolated vs combined), BMI (<25 kg/m^2^ vs ≥25 kg/m^2^),^28^ and time from meniscectomy (<5 years vs ≥5 years)^17^ as independent variables. The same variables were used in logistic regression analysis models when assessing patients not achieving the PASS for the various scores; the results were reported as odd ratios with 95% CI. Multiple and logistic regression analyses were normalized for the preoperative value of each score. Differences were considered significant with *P* < .05.

## Results

### Patient Characteristics

**Figure 1.**
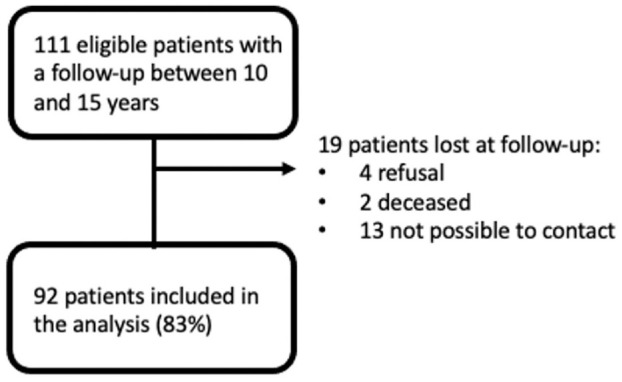
CONSORT (Consolidated Standards of Reporting Trials) diagram.

A total of 111 patients met the inclusion criteria, and 92 (83%) patients completed the long-term assessment at a mean follow-up of 11.8 ± 1.8 years (Figure 1). In the study population, there were 70 men (76%) and 22 women (24%) with a mean age at surgery of 42.2 ± 10.8 years. A total of 68 patients received medial CMI (74%) while 24 received lateral CMI (26%) after a mean of 9.7 ± 8.9 years from the meniscectomy (Table 2). A total of 36 cases (39%) were “isolated” while 56 cases (61%) were “combined” procedures. In the study population, 19 patients (21%) underwent acute CMI implantation (within 6 months from the previous meniscectomy) and 14 (15%) patients underwent CMI as an isolated procedure. There were 24 patients (26%) who underwent an associated cartilage treatment (Table 2). Twelve cases were located on the medial femoral condyle, 4 on the medial tibial plateau, 6 on the lateral femoral condyle, 1 on the lateral tibial plateau, and 1 on the trochlea. The cartilage lesions were 1 to 2 cm^2^ in 6 patients, 2 to 3 cm^2^ in 10 patients, and >3 cm^2^ in 8 patients.

**Table 2:** Patient Demographics and Surgical Characteristics^*a*^ (n = 92)

Variable		Value, n (%)
Follow-up, y		11.8 ± 1.8
Sex	Men	70 (76)
	Women	22 (24)
Age at surgery, y	Mean (SD)	42.2 ± 10.8
	<45	58 (63)
	≥45	34 (37)
Body mass index, kg/m^2^	Mean (SD)	25.4 ± 3.4
	<25	44 (48)
	≥25	48 (52)
CMI side	Medial	68 (74)
	Lateral	24 (26)
Combined procedure	Total	56 (61)
	ACLR	25 (27)
	Primary	17
	Revision	8
	Knee osteotomy	21 (23)
	HTO	18
	DFO	3
	Cartilage treatment	24 (26)
	Microfracture	16
	Osteochondral scaffold	6
	Mosaicplasty	1
	ACI	1
	>1 concomitant procedure	29 (31)
Outerbridge grade	Median (IQR)	3 (0-3)
	0-2	40 (43)
	3-4	52 (57)
Time from meniscectomy, y	Mean (SD)	9.7 ± 8.9
	<5	40 (43)
	≥5	52 (57)

a ACI, autologous chondrocyte implantation; ACLR, anterior cruciate ligament reconstruction; CMI, collagen meniscal implant; DFO, distal femoral osteotomy; HTO, high tibial osteotomy; IQR, interquartile range. Values presented as n (%), unless otherwise indicated.

### Clinical Outcomes

A total of 25 patients (27%) underwent a surgical procedure during the considered follow-up, at a mean of 4.3 ± 4.0 years from the CMI. Of these, only 12 cases (13%) were considered surgical failures (Table 3). Moreover, 19% of patients did not achieve the PASS for the KOOS Pain subscale, 40% for the KOOS Symptoms subscale, 23% for the KOOS ADL subscale, 24% for the KOOS Sport subscale, and 36% for the KOOS QoL subscale.

**Table 3:** Reoperations and Failures^*a*^

Variable	Value, n (%)
Total reoperations	25 (27)
Mean time to reoperation, y	4.3 ± 4.0
Failures	12 (13)
Mean time to failure, y	4.3 ± 4.0
MAT	4 (4)
UKA	3 (3)
TKA	3 (3)
CMI removal (infection)	1 (1)
CMI removal (HTO)	1 (1)
Other reoperations	13 (14)
Mean time to other reoperation, y	4.2 ± 4.1
Hardware removal	7 (8)
ACL reconstruction	3 (3)
ACL tunnel cyst removal	1 (1)
Arthroscopic debridement	1 (1)
Stem cell injection	1 (1)

a Values are presented as mean ± SD or n (%).ACL, anterior cruciate ligament; CMI, collagen meniscal implant; HTO, high tibial osteotomy; MAT, meniscal allograft transplantation; TKA, total knee arthroplasty; UKA, unicompartmental knee arthroplasty.

A significant improvement of the Lyholm score was reported from 55.6 ± 17.2 preoperatively to 76.3 ± 19.6 at final follow-up (*P* = .0001). Overall, 17% of patients did not achieve the PASS for Lysholm. Considering surgical failures and patients who did not reach the PASS for Lysholm score, a total of 28 cases (30%) were considered clinical failures.

**Figure 2.**
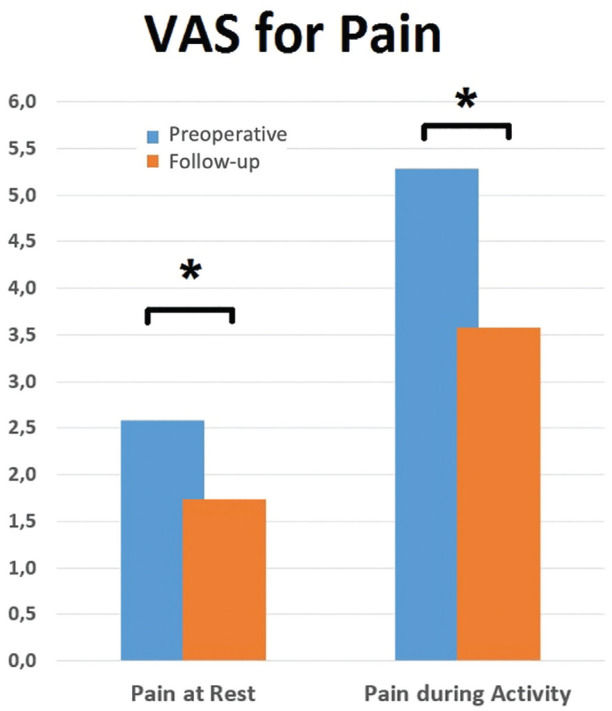
Comparison of pain at rest and pain during activity preoperatively and postoperatively. *Statistically significant at *P* < .005. VAS, visual analog scale.

**Figure 3.**
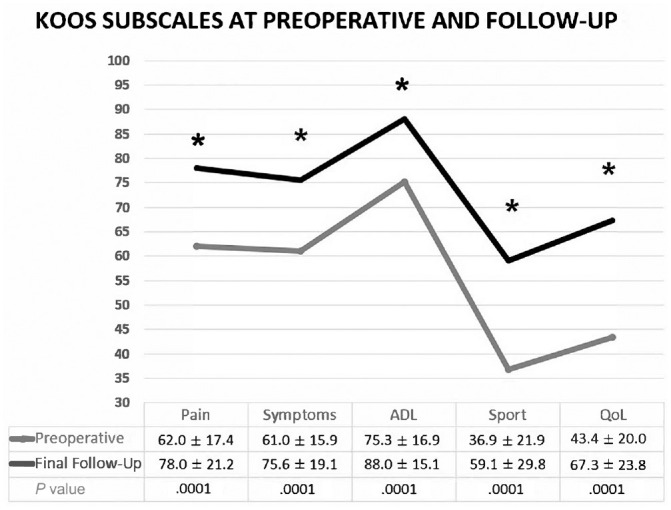
Knee injury and Osteoarthritis Outcome Score (KOOS) subscales preoperatively and postoperatively. *Statistically significant at *P* < .005. ADL, Activities of Daily Living; QoL, Quality of Life; Sport, Sport and Recreation.

The Tegner score improved from 4 (interquartile [IQR], 3-5) preoperatively to 5 (IQR, 4-5) at final follow-up (*P* = .002), not reaching the preinjury level of 7 (IQR, 6-8). The VAS for pain significantly decreased over time for both pain at rest (*P* = .008) and during activity (*P* = .0001) (Figure 2), and the mean KOOS scores improved for all subscales as well (Figure 3).

A total of 61% of patients were still practicing sports at the final follow-up, with 41% at the same or higher level. In particular, 34 patients were involved in pivoting sports (such as basketball, soccer, tennis, skiing), 8 patients were involved in hiking or trekking activities, 7 patients were still swimming, 3 patients were cycling, 1 patient was still involved in triathlon and 3 patients were practicing other sports. The overall mean satisfaction was 4.0 out of 5.0, and 80% of patients were satisfied by the procedure.

### Isolated Versus Combined CMI

Demographics and surgical characteristics were similar between the patients with isolated CMI (n = 36) and those with a combined procedure (56 patients), except for the degree of chondropathy and the time since meniscectomy, which were significantly higher in the case of combined procedures (Table 4).

**Table 4:** Demographics of Patients With Isolated CMI Versus CMI With Combined Procedures^*a*^

Demographic	Isolated (n = 36)	Combined (n = 56)	*P*
Sex (male/female)	28 (78)/8 (22)	42 (75)/14 (25)	.8074
Age at surgery, y	44.3 ± 11.7	40.8 ± 10.1	.1455
Follow-up, y	12.5 ± 2.0	11.3 ± 1.8	.6198
BMI, kg/m^2^	25.1 ± 2.5	25.6 ± 3.7	.4781
CMI side (medial/lateral)	27 (75)/9 (25)	41 (73)/15 (27)	.8576
Outerbridge grade			
Median (IQR)	0 (0-3)	3 (2-4)	**.0231**
0-2	22 (61)	18 (32)	
3-4	14 (39)	38 (68)	**.0094**
Time from meniscectomy, y			
Mean ± SD	6.2 ± 7.6	12.1 ± 9.1	**.0017**
<5	21 (58)	19 (34)	
≥5	15 (42)	37 (66)	**.0308**

a Values are presented as mean ± SD or n (%), unless otherwise indicated. BMI, body mass index; CMI, collagen meniscal implant; IQR, interquartile range.

**Figure 4.**
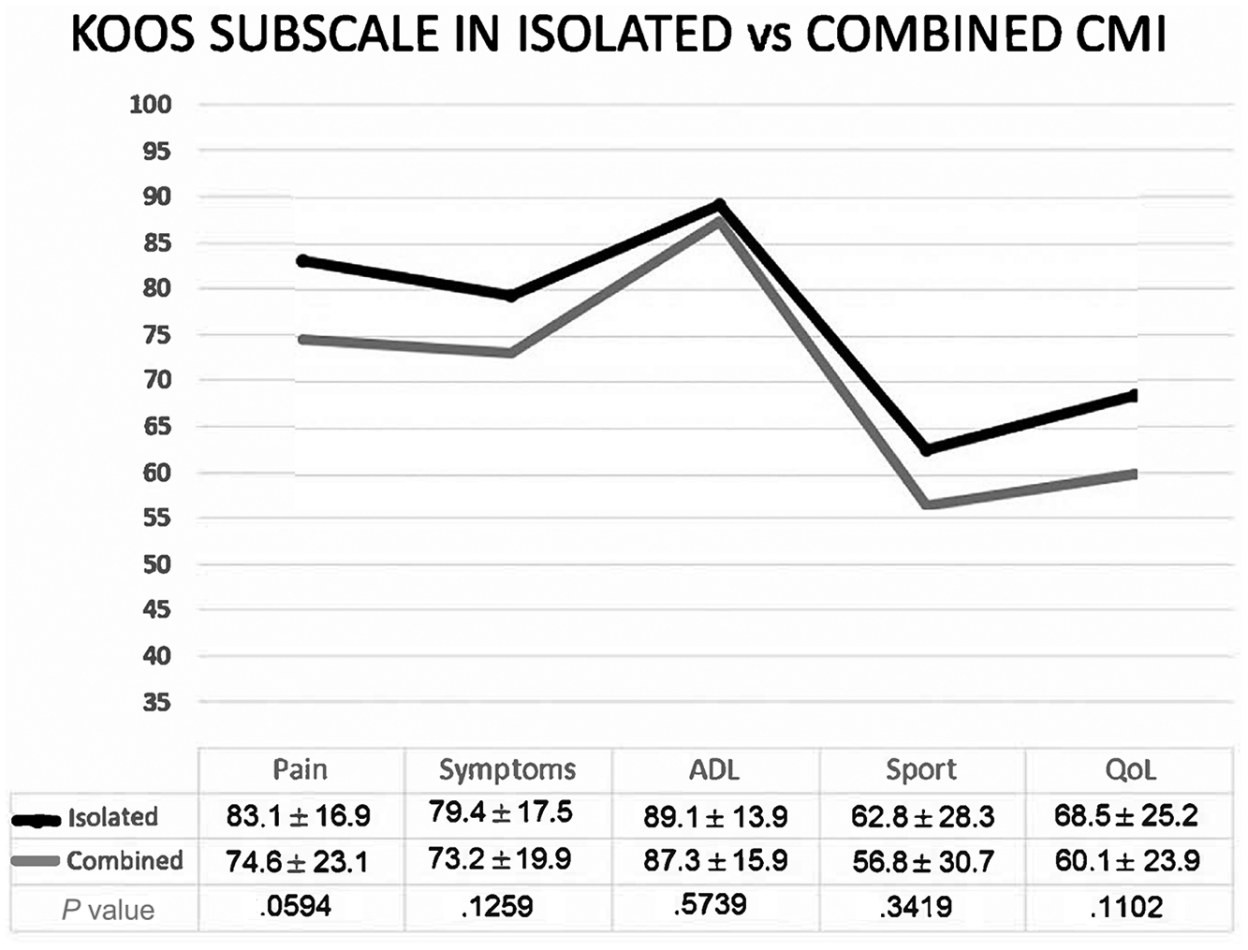
A comparison of isolated and combined CMI based on the KOOS subscale. ADL, Activities of Daily Living; CMI, collagen meniscal implant; KOOS, Knee injury and Osteoarthritis Outcome Score; QoL, Quality of Life; Sport, Sport and Recreation.

Regarding clinical outcomes, significant improvements of all patient-reported outcome measures were reported in both isolated and combined groups (Table 5). No significant differences were seen in patient-reported outcome measure assessment between the 2 groups, and a higher postoperative proportion of patients achieved PASS for the KOOS Pain subscale in the isolated group at the follow-up (Figure 4).

**Table 5:** Outcomes of Patients With Isolated CMI Versus CMI With Combined Procedures^*a*^

	Isolated (n = 36)	Combined (n = 56)	*P*
Outcomes			
Lysholm			
Preoperative	60.7 ± 17.6	52.3 ± 16.2	**.0207**
Follow-up	78.8 ± 17.4	74.6 ± 20.9	.3233
Improvement	18.1 ± 19.0	22.4 ± 23.8	.3212
Achieving PASS (>66.0)	26 (72)	41 (73)	**<.001**
VAS for pain (rest)			
Preoperative	2.1 ± 1.8	2.9 ± 1.7	.0340
Follow-up	1.2 ± 1.9	2.1 ± 2.3	.0525
Improvement	0.8 ± 2.5	0.6 ± 1.8	.6568
VAS for pain (activity)			
Preoperative	4.9 ± 2.4	5.5 ± 1.9	.1862
Follow-up	3.0 ± 2.6	4.0 ± 2.9	.0965
Improvement	1.5 ± 3.4	1.3 ± 2.3	.7371
KOOS Pain			
Preoperative	65.3 ± 17.5	60.0 ± 17.1	.1614
Follow-up	83.1 ± 16.9	74.6 ± 23.1	.0594
Improvement	17.9 ± 21.4	14.6 ± 22.7	.5560
Achieving PASS (>43.0)	30 (83)	41 (73)	**.0124**
KOOS Symptoms			
Preoperative	66.1 ± 12.3	57.8 ± 16.7	**.0153**
Follow-up	79.4 ± 17.5	67.8 ± 19.9	.1259
Improvement	13.3 ± 16.5	20.0 ± 20.8	.5272
Achieving PASS (>73.0)	25 (69)	31 (55)	.1963
KOOS ADL			
Preoperative	74.7 ± 13.7	75.6 ± 17.1	.7965
Follow-up	89.1 ± 16.9	87.3 ± 15.9	.5793
Improvement	14.4 ± 15.0	11.7 ± 20.8	.5181
Achieving PASS (>74.5)	30 (83)	41 (73)	.3150
KOOS Sport			
Preoperative	40.1 ± 23.0	34.8 ± 21.1	.2616
Follow-up	62.8 ± 28.3	56.7 ± 30.7	.3419
Improvement	22.6 ± 26.2	21.9 ± 30.9	.9745
Achieving PASS (>22.5)	31 (86)	39 (70)	.0838
KOOS QoL			
Preoperative	48.5 ± 22.1	40.2 ± 18.0	.0523
Follow-up	68.6 ± 25.2	60.1 ± 23.9	.1102
Improvement	20.0 ± 28.7	19.9 ± 23.4	.8126
Achieving PASS (>53.0)	27 (75)	32 (57)	.1185
Reoperations	5 (14)	20 (36)	**.0300**
Surgical failures	2 (6)	10 (18)	.1171
Clinical failures	10 (28)	18 (32)	.8168

a Values are presented as mean ± SD or n (%). Significative *p* values are in bold (*p* > 0.05) ADL, Activities of Daily Living; CMI, collagen meniscal implant; KOOS, Knee injury and Osteoarthritis Outcome Score; PASS, Patient Acceptable Symptom State; QoL, Quality of Life; Sport, Sport and Recreation; VAS, visual analog scale.

A significantly higher proportion of reoperations was reported in the combined group (36%) with respect to the isolated group (14%); however, no difference between the 2 groups was reported for clinical and surgical failures despite higher rates reported in the case of a combined procedure (Table 5).

### Assessment of Outcome Predictors

**Figure 5.**
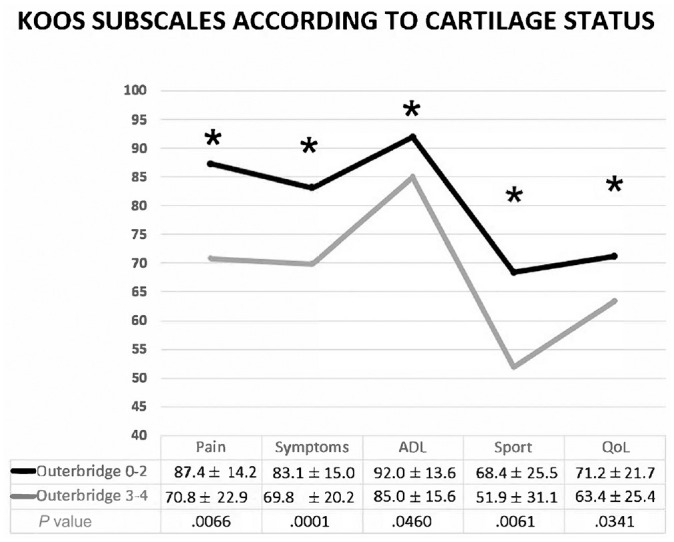
Comparison of Outerbridge grades 0 to 2 and grades 3 to 4 according to cartilage status. *Statistically significant difference between the groups (*P* < .05). ADL, Activities of Daily Living; KOOS, Knee injury and Osteoarthritis Outcome Score; QoL, Quality of Life; Sport, Sport and Recreation.

According to the multiple regression analysis performed on the whole series, having a chondropathy of Outerbridge grades 3 to 4 resulted in −16.5 points in the KOOS Pain subscale, −11.3 points in the KOOS Symptoms subscale, −7.0 points in the KOOS ADL subscale, −15.6 points in the KOOS Sport subscale, −8.4 points in the Lysholm score, +1.4 points in VAS pain at rest and +2.6 points in VAS pain during activity (Table 6); moreover, significantly lower values were reported in patients with chondropathy grades 3 to 4 compared with those with chondropathy grades 0 to 2 (Figure 5). Another outcome predictor was the timing of CMI of ≥5 years after the meniscectomy, which resulted in +0.9 points in VAS pain at rest and −12.3 points in the KOOS QoL subscale.

**Table 6:** Multivariate Analysis of Patient-Reported Outcome Measures^*a*^

	Lysholm	Tegner	VAS Rest	VAS Activity	KOOS Pain	KOOS Symptoms	KOOS ADL	KOOS Sport	KOOS QoL
Sex (female)	N.S.	N.S.	N.S.	N.S.	N.S.	N.S.	N.S.	N.S.	N.S.
Age (≥45 y)	N.S.	N.S.	N.S.	N.S.	N.S.	N.S.	N.S.	N.S.	N.S.
Overweight (BMI ≥25 kg/m^2^)	N.S.	N.S.	N.S.	N.S.	N.S.	N.S.	N.S.	N.S.	N.S.
CMI side (lateral)	N.S.	N.S.	N.S.	N.S.	N.S.	N.S.	N.S.	N.S.	N.S.
Associated procedure (yes)	N.S.	N.S.	N.S.	N.S.	N.S.	N.S.	N.S.	N.S.	N.S.
Chondropathy (Outerbridge grades 3-4)	**–8.4 (*P* = .0402)**	N.S.	**+1.4 (*P* = .0008)**	**+2.6 *(P* = .0001)**	**–16.5 (*P* = .0001)**	**–11.3 (*P* = .0020)**	**–7.0 (*P* = .0202)**	**–15.6 (*P* = .0066)**	N.S.
Timing of CMI (≥5 y)	N.S.	N.S.	**+0.9 (*P* = .0441)**	N.S.	N.S.	N.S.	N.S.	N.S.	**–12.3 (*P* = .0075)**

a Multivariate analysis of the effect on patient-reported outcomes of patients and surgical characteristics. Significative *p* values are in bold (*p* > 0.05) ADL, Activities of Daily Living; BMI, body mass index; CMI, collagen meniscal implant; KOOS, Knee injury and Osteoarthritis Outcome Score; N.S., nonsignificant; QoL, Quality of Life; Sport, Sport and Recreation; VAS, visual analog scale.

According to the logistic regression analysis, having a chondropathy of Outerbridge grades 3 to 4 resulted in a higher risk of not achieving the PASS for all the KOOS subscales and Lysholm, while an age at surgery of ≥45 years resulted in a lower risk of not achieving PASS for the KOOS Pain subscale (Table 7).

**Table 7:** Analysis of PASS for KOOS and Lysholm Scores^*a*^ (n = 92)

		KOOS Pain		KOOS Symptoms		KOOS ADL		KOOS Sport		KOOS QoL		Lysholm	
		No.	(%)	No.	(%)	No.	(%)	No.	(%)	No.	(%)	No.	(%)
Patients achieving PASS threshold													
Yes		74	(80)	55	(60)	70	(76)	69	(75)	58	(63)	67	(73)
No		17	(19)	36	(39)	21	(23)	22	(24)	33	(36)	22	(24)
Logistic regression for not achieving PASS		OR (95% CI)	*P*	OR (95% CI)	*P*	OR (95% CI)	*P*	OR (95% CI)	*P*	OR (95% CI)	*P*	OR (95% CI)	*P*
Sex (female)		N.S.		N.S.		N.S.		N.S.		N.S.		N.S.	
Age (≥45 y)		0.2 (0.1-0.8)	.02889	N.S.		N.S.		N.S.		N.S.		N.S.	
Overweight (BMI ≥25 kg/m^2^)		N.S.		N.S.		N.S.		N.S.		N.S.		N.S.	
CMI side (lateral)		N.S.		N.S.		N.S.		N.S.		N.S.		N.S.	
Associated procedure (yes)		N.S.		N.S.		N.S.		N.S.		N.S.		N.S.	
Chondropathy (Outerbridge grades 3-4)		29.2 (3.1-270.2)	.0029	4.5 (1.7-12.1)	.0031	4.6 (1.4-15.6)	.0138	8.8 (2.2-35.7)	.0022	5.0 (1.8-14.0)	.0024	3.7 (1.2-11.2)	.0200
Timing of CMI (≥5 y)		N.S.		N.S.		N.S.		N.S.		N.S.		N.S.	

a Significative *p* values are in bold (*p* > 0.05) ADL, Activities of Daily Living; BMI, body mass index; CMI, collagen meniscal implant; KOOS, Knee injury and Osteoarthritis Outcome Score; N.S., nonsignificant; OR, odds ratio; PASS, Patient Acceptable Symptom State; QoL, Quality of Life; Sport, Sport and Recreation.

## Discussion

The most important finding of the present study is that 70% of patients achieved satisfactory clinical results, whereas 13% of cases were surgical failures and another 17% had intact implants but poor clinical scores. The sports activity level steadily improved compared with the preoperative status, and almost two-thirds of the patients were still active at the long-term evaluation. Furthermore, several factors were associated with lower clinical scores, including an increasing time from meniscectomy to scaffold implantation, age, and Outerbridge grade.

Satisfactory clinical outcomes of the meniscal scaffold implantation have been previously reported, with an improvement in symptoms and function compared with the preoperative status at short- to midterm follow-up.^20^ However, the authors reported that only 3 case series investigated the long-term outcomes of CMI, and none of the cases evaluated the PASS and the predictive factors affecting the clinical outcomes after meniscal scaffold substitution.

Two long-term case series with <40 patients reported significant and stable clinical outcomes at a 10-year follow-up after medial meniscal scaffold implantation with only 2 overall failures.^18,27^ On the other hand, results from a long-term multicenter study on the lateral CMI reported a significantly higher failure rate, more pain, and a lower activity level when compared with the results in existing literature on the medial scaffold.^11^ Similarly, a recent long-term survival analysis identified the lateral scaffold as a risk factor for surgical failure, with a 10-year survival rate of 77% versus 90% of the medial CMI.^17^

Conversely, in the present study, the multivariate analysis found no significant difference in patient-reported outcome measures concerning the medial versus lateral scaffold. Those results align with a recent CMI and polyurethane meniscal scaffold meta-analysis that found no differences in clinical outcomes or survival rates between medial and lateral meniscal scaffolds at short- or midterm follow-up.^19^ Moreover, 70% of the patients achieved the PASS for Lysholm score; this could be considered a satisfactory result if we consider that meniscal substitution is usually performed on patients affected by postmeniscectomy syndrome and limited alternative procedures to joint replacement. The results reported in the present study are in line with a clinical study investigating the outcomes of MAT at a similar follow-up time, with a Lysholm score around 80 points at 10-year follow-up.^10^

In the current study, the return-to-sports rate, reduced pain during activity, and Tegner level at the final follow-up should be considered satisfactory considering the mean age of the patients and the multiple knee comorbidities of the study population. Similar to studies investigating the outcomes of MAT, return-to-sports activity appears to be a reasonable expectation after meniscal scaffold substitution, especially for low-impact sports.^9^

However, because there are few studies and limited evidence about the impact of sports activity and cartilage degeneration after meniscal substitution, high-impact activities must be considered with caution, keeping in mind that the general ambition should be to preserve the knee function for ADLs and work.^20,21,23,24^

An interesting finding of the present study is that patients who underwent isolated CMI showed similar overall outcomes but less knee pain when compared with patients who underwent combined surgeries. These results contrast with those of Gelber et al,^7^ who reported inferior clinical results in patients with varus knee and meniscal deficiency when the high tibial osteotomy was associated with the implant of a polyurethane meniscal scaffold.

Our findings further underline the effect of the CMI as a standalone procedure and contest the conclusion of a recent systematic review which hypothesized that the presence of concomitant surgeries may have a significant influence on the positive results after scaffold substitution.^13^ However, considering that 14 out of 36 patients (39%) included in the isolated group underwent acute CMI implantation, it could also be hypothesized that the clinical benefit could be related to the meniscectomy itself rather than the scaffold. For this reason and due to the heterogeneity of the study population, it remains challenging to draw a definitive conclusion regarding the effect of the isolated CMI.

In the present study, sex and BMI did not show any impact on the clinical outcomes. However, while the effect of sex has been less investigated in the meniscal scaffold literature, 2 separate reports showed a higher incidence of revision and surgical failures in female patients requiring MAT.^6,10^ Moreover, in contrast to our results, a higher BMI was correlated with inferior clinical results in a multicentric study of 43 patients who underwent lateral CMI.^26^ Thus, the effect of these 2 parameters on the clinical outcomes should be further investigated due to inconclusive evidence.

One of the most debated topics in the meniscal substitution literature is the ideal time elapse between meniscectomy and meniscal transplant or scaffold implant.^8^ In the present study, we found that patients who waited ≥5 years for meniscal scaffold substitution reported significantly higher pain scores and reduced QoL at the final follow-up.

In a large case series of CMI, the authors reported that waiting >10 years after meniscectomy was correlated with a significantly higher risk of surgical or clinical failure regardless of the cartilage status.^16^ Another study reported that waiting for as little as 6 months after a meniscectomy may reduce clinical outcomes and sports level at short-term follow-up.^2^ Finally, a study on the effect of meniscal transplant found that an acute MAT achieved better cartilage and meniscal protection in the long term compared with the conventional delayed MAT. However, the impact on clinical outcomes was minimal.^25^ Although some of the studies mentioned above found better clinical outcomes if a meniscal substitution procedure was performed in an acute setting, little is known about risk factors for developing postmeniscectomy syndrome. Therefore, further research is needed to help the surgeon identify the patients who may benefit from an acute scaffold or MAT surgery.

Another interesting result of the present study was that patients ≥45 years old not only showed similar outcomes when compared with younger patients but were also more likely to achieve the PASS in the KOOS Pain subscale. A previous study showed that age was not correlated with surgical failure.^22^ The present study’s clinical results further confirm that age does not represent a contraindication for meniscal scaffold. These data appear promising, considering that one of the most common patient profiles of the candidates for meniscal scaffold is represented by a middle-aged man with a previous partial meniscectomy or with meniscal tears on degenerated tissue. During scaffold substitution, the patient should be carefully evaluated. Clinical outcomes and survivorship are predominantly affected by age-related factors, including time from meniscectomy and chondropathy, rather than age itself.^22^ This is similar to the MAT.

Despite the strength of a long-term minimum follow-up of 10 years, the large number of patients, and the satisfactory follow-up rate, the present study presents several limitations. First, this is a retrospective study that lacks a control group of patients who underwent isolated meniscectomy or meniscal transplant. Moreover, the patients are heterogeneous in terms of age, sex, chondropathy, and concomitant procedures, making it more difficult to predict the outcomes of a specific population. However, the presence of multiple knee comorbidities represents a typical scenario of patients referred to a tertiary sports medicine center. Therefore, multiple subanalyses were performed to evaluate each parameter individually. Additionally, the PASS used in the present analysis was validated for patients requiring MAT.^15^ Nevertheless, it is to be considered that there are no PASS scores validated for meniscal scaffold and that the demographic characteristics and knee pathology of patients requiring 1 of either of these 2 procedures are similar. Last, since no magnetic resonance imaging was performed at the last follow-up, it was not possible to evaluate parameters such as cartilage degeneration over time, scaffold extrusion, or reabsorption. However, this study was specifically designed to evaluate predictors of clinical outcomes and the radiological classification system. Features of meniscal implant have been shown to be poorly correlated with a patient’s symptoms, especially at a long-term follow-up.^12-14^

## Conclusion

Up to 10 years after surgery, around 70% of the patients who underwent CMI reported satisfactory clinical results, with subjective scores still higher compared with the preoperative evaluation. A total of 28 cases (30%) were classified as clinical failures (13% surgical failure and 17% not meeting PASS for Lysholm score). In addition, the cartilage status and the time from meniscectomy were shown to have a negative impact on outcomes, while an age ≥45 years was associated with less pain. Finally, there was no clinical difference between patients who underwent isolated CMI or combined procedures.
